# Perception of Korean healthy adolescents on cancer and adolescent cancer survivors: a cross-sectional survey

**DOI:** 10.1186/s12889-024-19192-4

**Published:** 2024-06-26

**Authors:** Min Kyung Hyun, Yeonseung Lee, Hyun Jeong Lee, Young Ae Kim

**Affiliations:** 1 Department of Preventive Medicine, College of Korean Medicine, Dongguk University, Gyeong Ju, Republic of Korea; 2 Division of National Cancer Survivorship Center, National Cancer Center, Goyang‐si, Republic of Korea; 3 Division of Cancer Control & Policy, National Cancer Control Institute, National Cancer Center, Goyang-Si, Republic of Korea

**Keywords:** Perception, Cancer survivors, Adolescent, Cross-sectional studies

## Abstract

**Background:**

As the number of adolescent cancer survivors increases, detailed and effective healthcare policies on adolescent cancer survivors returning to school and workplace are needed. The study aimed to explore the perception of healthy adolescents on cancer and adolescent cancer survivors.

**Methods:**

This study conducted a face-to-face cross-sectional study in the Republic of Korea in 2021 on adolescent selected through proportional population allocation sampling by sex, age, and region. According to research questions, survey questionnaire organized and collected data on adolescents' perceptions of cancer, differences in perceptions from tuberculosis, measles, asthma, perceptions of adolescent cancer survivors, and health information sources that led to these perceptions.

**Results:**

Of the total 500 adolescents, less than 10% of healthy adolescents responded that cancer is contagious, while three-quarters of the respondents believed that cancer is preventable. In addition, compared to tuberculosis, measles, and asthma, they recognized differences by disease. The majority of healthy adolescents embraced community values advocating the return of adolescent cancer survivors to school and work. However, they expressed a negative view of the situation in which adolescent cancer survivors could interact with them as classmates or co-workers. Adolescents mainly obtained health information on cancer from the Internet and television,

**Conclusions:**

The perception of healthy adolescents on cancer was relatively accurate; however, they have dualistic thinking involving living with adolescent cancer survivors. To facilitate reintegration of adolescent cancer survivors into daily lives, education is needed for healthy adolescents to live with cancer survivors.

**Supplementary Information:**

The online version contains supplementary material available at 10.1186/s12889-024-19192-4.

## Background

A study analyzing the Global Burden of Disease 2019 Study revealed that approximately 1,335,100 new cancer cases and 397,583 cancer-related deaths in adolescents and young adults worldwide were reported in 2019; interestingly, while the incidence of cancer slightly increased, a significant decline in the mortality rate was observed [1]. In 2024, 4.2% of all new cases is projected to occur between the ages of 15 and 39 years, with 85.9% of them surviving cancer for five years after diagnosis based on the new estimate from the National Cancer Institute in the United States [2]. Meanwhile, the projected survival rate for those aged 0–14, 40–64, and ≥ 65 years is 85.1%, 74.1%, and 61.5%, respectively [3]. In a study analyzing the registration data of the Korea Central Cancer Registry in Republic of Korea (ROK), 10,476 adolescents aged 15–19 years had cancer between 1999 and 2016; the age-standardized incidence rate for those aged 15–19 years was 170.4 per million, and the degree of survival improvement was the highest in this age group [4]. Data worldwide, including the United States and ROK reveal two key facts about adolescent cancer: while diagnoses are less frequent compared to the overall cancer population, the survival rates are relatively high.

In addition to concerns and management of their ongoing or potential health issues, adolescent cancer survivors face tasks common to of their peers, such as navigating the social landscape of school, building a career path towards independent adulthood, and finding their place as contributing members of society [5–8]. Prior studies have indicated that adolescent cancer survivors who experience difficulties returning to normal life may be at increased risk of unemployment and social isolation [8–11]. A study conducted in ROK on 145 adolescent cancer survivors aged 13–18 years revealed that 49.7% reported experiencing at least one type of peer exclusion and victimization in school [12]. Thus, paying attention to the specific needs and support system for adolescent cancer survivors is crucial. Accordingly, several countries have provided various support programs in addition to clinical treatment for adolescent patients with cancer [5, 6] Studies on the perceptions of healthy adolescent regarding cancer and adolescent cancer survivors, particularly in school or in workplace, remain limited [13, 14].

Given these circumstances, conducting studies that addresses the lack of understanding among healthy adolescents regarding cancer diseases and adolescent cancer survivors is imperative. Thus, we aimed to explore the perception of healthy adolescents regarding cancer, determine if these perceptions differ from those of other diseases, understand the perception of adolescent cancer survivors, and identify health information resources.

## Methods

### Study design and questionnaire

This was a cross-sectional study conducted from September 13 to October 7, 2021. Since there were no existing reports or an officially developed questionnaires that met the research objectives in ROK, the questionnaire was developed based on a literature review and expert advice. The perception items and the diseases to be compared to cancer were mainly based on the items from the 2008 and 2018 cancer perception surveys conducted in Japan for adolescents aged 10–15 years [15].

The questionnaire comprised the following components: 1) demographic information including sex, school grade level, region of residence, birth order, smoking experience, drinking experience, satisfaction with school life, self-rated health, and level of stress, 2) adolescents’ perception of cancer and other diseases such as tuberculosis, measles, and asthma, 3) adolescents’ perception of adolescent cancer survivors, and 4) sources of health information, such as television, internet, book, teacher, parents, siblings, and friends. The perception items related to the disease in the questionnaire consisted of contagious, affecting adolescents, preventable, serious, and curable. The adolescents' perception of adolescent cancer survivors was divided into three domains: general, being classmates, and being co-workers. Within each domain, 3–4 questions were organized to assess the respective perceptions.

### Data collection methods

The survey participants were healthy adolescents aged 14–18 years without prior cancer diagnosis. A non-probability sampling method was employed, where sample sizes were allocated based on the population proportions of sex, age, and region according to the Ministry of the Interior and Safety’s April 2021 resident registration population statistics. Participants were categorized by sex (male and female) and age (14–18 years old, born between 2003 and 2007) into five 1-year-age-groups. The sampling frame encompassed 17 regions: Seoul, Incheon, Busan, Daegu, Gwangju, Daejeon, Ulsan, Sejong, Jeju-do, Gyeonggi-do, Gangwon-do, Chungcheong Buk-do, Chungcheong Nam-do, Joella Buk-do, Joella Nam-do, Gyeong Sang Buk-do, and Gyeong Sang  Nam-do.

To recruit participants, middle and high schools in these 17 regions were contacted, and permission for student participation was obtained. The survey was subsequently conducted after securing both student and parental consent. Of the 887 individuals invited to participate, 500 agreed (resulting in a 56.4% response rate). The main reasons for not participating in the survey were parental refusal and adolescents' own refusal due to various concerns. A one-on-one, face-to-face survey was then conducted with the consenting participants.

Finally, the survey was completed on 100 second graders (14 years old) and 94 third graders (15 years old) in middle school, as well as 97 first graders (16 years old), 103 second graders (17 years old), and 106 third graders (18 years old) in high school.

The distribution of survey participants by region is detailed in Appendix 1, and regional data were analyzed by dividing 17 regions into three types: Seoul capital area (Seoul, Gyeonggi, and Incheon), metropolitan city (Busan, Daegu, Gwangju, Daejeon, Ulsan, Sejong, and Jeju), and other regions.

### Statistical analysis

Baseline characteristics of the participants were summarized using descriptive statistics. The chi-square test was used to examine differences between categorical variables, such as demographic information and adolescents’ perception of adolescent cancer survivors. The student’s t-test was used to compare the means of continuous variables (e.g., self-rated health and level of stress) between two groups defined by sex (male vs. female) and education stage (middle vs. high school). For categorical variables with three or more groups (e.g., adolescents’ perception of cancer, asthma, measles, and tuberculosis, and health information resources), ANOVA was used to compare the means among the groups. We analyzed the data for all participants, stratified by sex and education stage, to investigate our hypothesis that perception might differ based on these factors.

All data manipulation, which refers to the process of preparing and organizing data for statistical analysis, and statistical analyses were conducted using Stata/MP version 17 (Stata Corp LP, College Station, TX, USA). A *p* < 0.05 was considered statistically significant.

## Results

### Characteristics of the respondents

Of the 500 adolescents, 260 were men and 240 were women. Moreover, 194 and 306 were middle school (15–16 years old) and high school students (17–19 years old), respectively. Among the participants, only child accounted for 11.80% while the firstborn for 44.20%. When analyzing by sex and education stage, a statistically significantly difference was observed between smoking and drinking. Additionally, a statistically significant difference was found in stress levels measured using the stress 0–10 visual analogue scale between middle and high school students (Table 1).

**Table 1:** Characteristics of the respondents (*n* = 500)

Variables	Total (*n* = 500)		Male (*n* = 260, 52%)		Female (*n* = 240, 48%)		*P*	Middle school (*n* = 194, 38.80%)		High school (*n* = 306, 61.20%)		*P*
	n	%	n	%	n	%		n	%	n	%	
Sex												0.982
Male	260	52.00						101	38.85	159	61.15	
Female	240	48.00						93	38.75	147	61.25	
School year (Age)							0.982					
Middle school (15–16)	194	38.80	101	52.06	93	47.94						
High school (17–19)	306	61.20	159	51.96	147	48.04						
Region							0.82					0.676
Seoul capital area	246	49.20	126	51.22	120	48.78		97	39.43	149	60.57	
Metropolitan city	112	22.40	57	50.89	55	49.11		46	41.07	66	58.93	
Other regions	142	28.40	77	54.23	65	45.77		51	35.92	91	64.08	
Birth order							0.594					0.513
Only child	59	11.80	30	50.85	29	49.15		26	44.07	33	55.93	
Firstborn	221	44.20	110	49.77	111	50.23		88	39.82	133	60.18	
Second or more child	220	44.00	120	54.55	100	45.45		80	36.36	140	63.64	
Smoking experience							**0.002**					**0.001**
No	409	81.80	199	48.66	210	51.34		173	42.30	236	57.70	
Yes	91	18.20	61	67.03	30	32.97		21	23.08	70	76.92	
Drinking experience							** < 0.001**					** < 0.001**
No	361	72.20	168	46.54	193	53.46		158	43.77	203	56.23	
Yes	139	27.80	92	66.19	47	33.81		36	25.90	103	74.10	
Satisfaction with school life							0.631					0.075
Unsatisfied	50	10.00	28	56.00	22	44.00		12	24.00	38	76.00	
Neutral	141	28.20	69	48.94	72	51.06		56	39.72	85	60.28	
Satisfied	309	61.80	163	52.75	146	47.25		126	40.78	183	59.22	
Encounter with Pediatric and Adolescent Cancer Survivors							0.208					0.547
No	487	97.40	251	51.54%	236	48.46%		190	39.01%	297	60.99%	
Yes	13	2.60	9	69.23%	4	30.77%		4	30.77%	9	69.23%	
	mean	SD	mean	SD	mean	SD		mean	SD	mean	SD	
Self-rated health (0–100 visual analogue scale)	82.59	11.95	82.90	11.98	82.25	11.92	0.544	81.85	12.98	83.06	11.24	0.270
Stress (0–10 visual analogue scale)	4.79	2.16	4.79	2.14	4.80	2.18	0.986	4.45	2.02	5.01	2.22	**0.004**

### Adolescents’ perception of cancer, asthma, measles, and tuberculosis

Of the respondents, 8.42% believed that cancer is contagious, 66.33% are aware of its effects on adolescents, 72.34% responded that cancer is preventable, 95.19% considered it as a serious disease, and 92.18% acknowledge it as treatable.

A difference in the perception of cancer and all other diseases in terms of their impact on adolescents and preventability were observed. Conversely, there were differences in the perception of contagiousness between cancer and measles, as well as asthma.

**Fig. 1:**
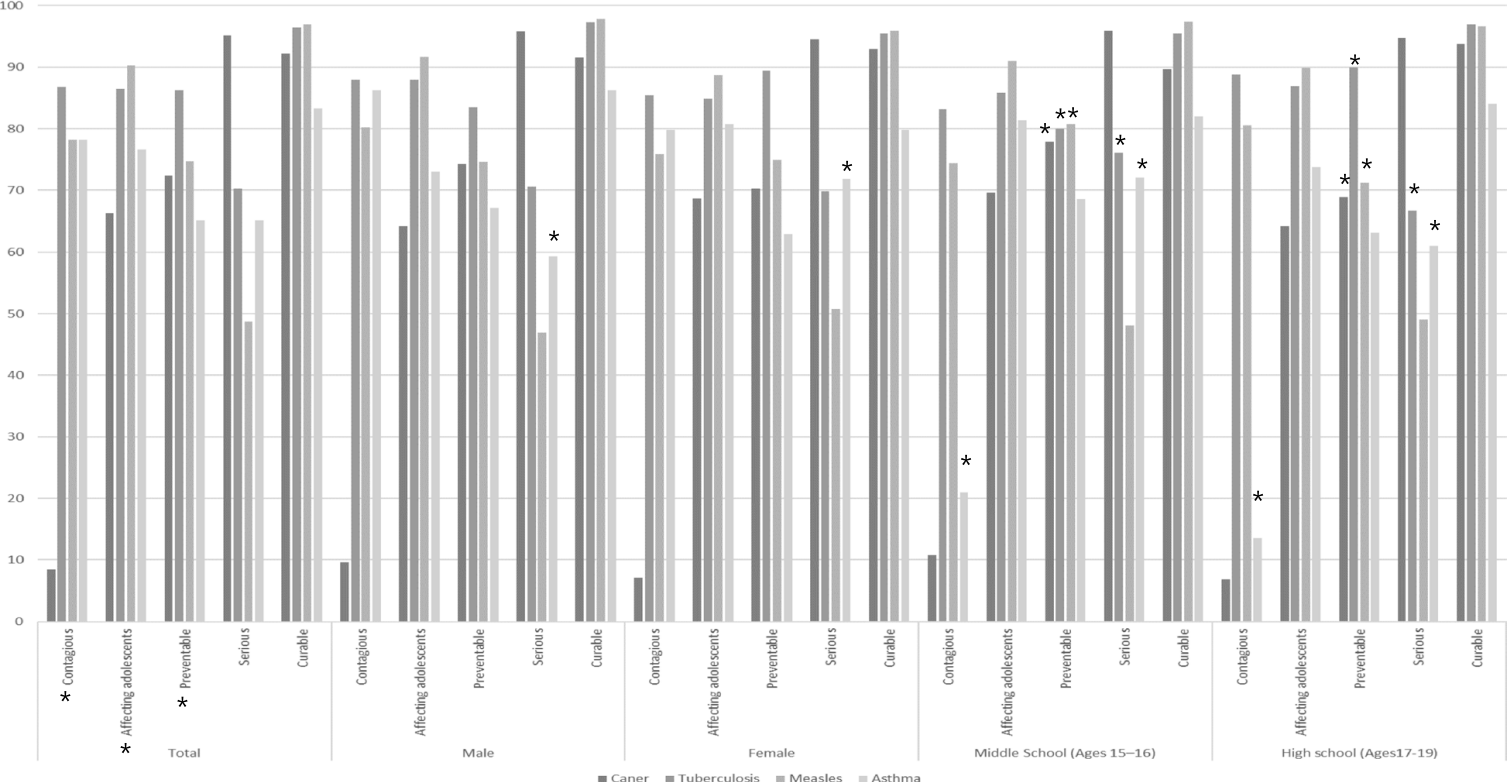
Adolescents' perception of cancer, asthma, measles, and tuberculosis by sex and education stage. * is statistically significant at *p* < 0.05

Only the perception of the severity of asthma and not of other diseases differed by sex. There were differences in perception between middle and high school students regarding the contagiousness of asthma, preventability of cancer, tuberculosis, and measles, as well as the severity of tuberculosis and asthma (Fig. 1, Appendices 2–4).

### Adolescents’ perception of adolescent *cancer* survivors

Of the respondents, 17.4% recognize the existence of adolescent cancer survivors, and only 7.6% expressed interest regarding this. About two-thirds of the respondents indicated that they would be uncomfortable being in the presence of adolescent cancer survivors. However, 82.8% of respondents agreed that society should protect adolescent cancer survivors.

Moreover, 48.60% of the respondents expressed support for studying with adolescent cancer survivors as a classmate, whereas 5.80% expressed opposition. More than half, or 52.8% of the respondents, responded that adolescent cancer survivors’ learning skills would be sluggish. However, 68.8% agreed that equal opportunities should be given to adolescent cancer survivors to participate in class activities.

The response towards being a co-worker was notably more extreme, as 81.4% of the respondents expressed a desire to avoid working with adolescent cancer survivors. Additionally, 48.4% of the respondents believed that it would be difficult for adolescent cancer survivors to secure employment or establish a successful work life. However, 91.6% acknowledge that adolescent cancer survivors can contribute to society. Furthermore, when analyzing the data by sex and education stage, a statistically significant difference was observed only in the level of interest shown towards adolescent cancer survivors between middle and high school students (Table 2).

**Table 2:** Adolescents’ perception of adolescent cancer survivors (*n* = 500)

Variables	Total (*n*-500)		Male (*n* = 260, 52%)		Female (*n* = 240, 48%)		*P*	Middle school (*n* = 194, 38.80%)		High school (*n* = 306, 61.20%)		*P*
	n	%	n	%	n	%		n	%	n	%	
**General**												
Recognize of existence							0.955					0.060
No	413	82.60	215	52.06%	198	47.94%		168	40.68%	245	59.32%	
Yes	87	17.40	45	51.72%	42	48.28%		26	29.89%	61	70.11%	
Interest in them							0.816					** < 0.001**
Absent	332	66.40	176	53.01%	156	46.99%		151	45.48%	181	54.52%	
Somewhat	130	26.00	65	50.00%	65	50.00%		33	25.38%	97	74.62%	
Present	38	7.60	19	50.00%	19	50.00%		10	26.32%	28	73.68%	
Uncomfortable being around them							0.185					0.815
Yes	181	36.20	87	48.07%	94	51.93%		69	38.12%	112	61.88%	
No	319	63.80	173	54.23%	146	45.77%		125	39.18%	194	60.82%	
Society needs to protect them							0.263					0.377
No	86	17.20	40	46.51%	46	53.49%		37	43.02%	49	56.98%	
Yes	414	82.80	220	53.14%	194	46.86%		157	37.92%	257	62.08%	
**Being classmates**												
Thoughts on studying with them							0.079					0.152
Opposed	29	5.80	13	44.83%	16	55.17%		10	34.48%	19	65.52%	
Neutral	228	45.60	131	57.46%	97	42.54%		99	43.42%	129	56.58%	
In favor	243	48.60	116	47.74%	127	52.26%		85	34.98%	158	65.02%	
Lacking of learning ability in them							0.758					0.121
Yes	264	52.80	139	52.65%	125	47.35%		94	35.61%	170	64.39%	
No	236	47.20	121	51.27%	115	48.73%		100	42.37%	136	57.63%	
Ensuring that they participate in classes							0.609					0.716
Disagree	17	3.40	10	58.82%	7	41.18%		5	29.41%	12	70.59%	
Neutral	139	27.80	76	54.68%	63	45.32%		55	39.57%	84	60.43%	
Agree	344	68.80	174	50.58%	170	49.42%		134	38.95%	210	61.05%	
**Being co-workers**												
Avoiding working with them							0.218					0.651
Yes	93	18.60	43	46.24%	50	53.76%		38	40.86%	55	59.14%	
No	407	81.40	217	53.32%	190	46.68%		156	38.33%	251	61.67%	
Expected difficulties in their employment or work life							0.386					0.474
Yes	242	48.40	121	50.00%	121	50.00%		90	37.19%	152	62.81%	
No	258	51.60	139	53.88%	119	46.12%		104	40.31%	154	59.69%	
Able to contribute to society							0.786					0.275
No	42	8.40	21	50.00%	21	50.00%		13	30.95%	29	69.05%	
Yes	458	91.60	239	52.18%	219	47.82%		181	39.52%	277	60.48%	

### Health information resources

Adolescents primarily obtained health information on cancer from the Internet (46.4%) and television (42.4%), which was higher compared to that for tuberculosis, measles, and asthma. The disparity in information acquisition methods between cancer and other diseases showed statistical significance.

**Fig. 2:**
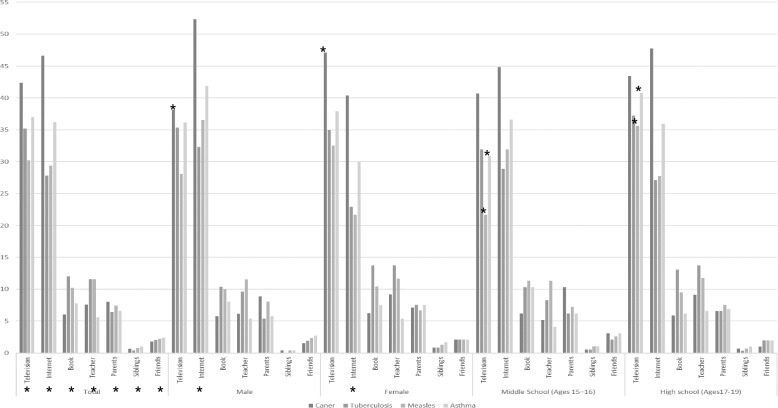
Health information resources by sex and education stage. * is statistically significant at *p* < 0.05

A sex differences were observed between men and women in obtaining health information on cancer, tuberculosis, measles, and asthma, particularly through the Internet. Additionally, differences were observed in obtaining information on cancer through the television. For measles and asthma, disparities were observed in television as a source of information between middle and high school students (Fig. 2, Appendices 5–7).

## Discussion

In this nationwide study, healthy adolescents demonstrated a relatively accurate perception of cancer and several diseases, based on health information resources such as television and internet. Furthermore, the majority of healthy adolescents embraced community values that advocated for the well-being of socially disadvantaged individuals, such as adolescent cancer survivors. However, they expressed negativity towards situations in which adolescent cancer survivors might interact with them as classmates or co-workers. In other words, healthy adolescents demonstrated dualistic thinking depending on whether the life of adolescent cancer survivors was directly relevant to them. Reports have surfaced warning about the negative perception of cancer survivors returning to work among the Korean adult population have already surfaced [16–18]. Our study further indicates that healthy adolescents also display similar tendencies. Therefore, changing the mindset of healthy adolescent by providing them accurate cancer survivorship education in formal settings such as schools is crucial. However, as of 2024, no health and disease-related education programs have been established in ROK other than that for smoking, drugs, and sexual violence prevention education, without a plan of opening for cancer survivorship in the future.

Despite both Korea and Japan being part of East Asia, a 2018 Japanese study on middle school students' perceptions of cancer revealed discrepancies with our findings for the same age group [15]. The study showed that more middle school students agreed on the effects of cancer on adolescents and its severity, while the rates of consent on contagiousness, preventability, and curability were lower than those in our study [15]. A big difference in health information resources was also observed between the two countries, with ROK’s main sources being the Internet and television, while that for Japan were television, parents, books, Internet, and teachers [15]. In ROK, 10.31% of middle student respondents stated that they obtained information on cancer from their parents, which was the highest among the surveyed diseases. In contrast, in Japan, middle school respondents have relatively diverse health information sources with approximately 70% coming from their parents, 60% from books and the Internet, and 40% from teachers [15]. The healthcare system in ROK is similar to that in Japan; however, significant differences were observed in the systematicity of school health education system, level of qualification required as an educator, and quantity and quality of education contents, which is believed to be a major contributor to the disparity in health information resources between the two countries [19]. Since 2017, the Japanese Ministry of Education, Culture, Sports, Science and Technology has initiated cancer education programs in schools, providing educational materials [15]. As a result, 56.3% of elementary schools and 71.4% of middle schools reportedly conducted classes on cancer education in 2018 [15]. While the focus of this education is primarily on adulthood cancer and its prevention and early detection, with limitations acknowledging that cancer could affect adolescent, it can be considered an improvement over ROK, which does not provide cancer education in schools.

Adolescent cancer survivors may encounter disease- or therapy-related late-effects, which limit their participation in various activities in their daily life, or even if they recover and become healthy adolescents, they continue to experience prejudices around them [5]. While there are limited studies on perceptions of healthy adolescents that directly compares to our study, stereotypes and stigma surrounding adolescent cancer survivors exist. In a study on the perception of cancer and infertility in healthy female adolescents published in 2013, participants expressed concern about potential infertility, hereditary transmission, and future effects from cancer treatment [20]. A systematic review in 2021 highlighted that adolescent cancer survivors may encounter challenges in social functioning influenced by various factors including negative body image, engagement in social comparisons, and the social/cultural stigma surrounding cancer [21]. Similar observations are also noted in parents and caregivers that closely care for cancer patients. In a study examining the perception of mothers with children diagnosed with cancer, mothers perceived their children as less independent compared to healthy children, and more often displayed an emotionally ambivalent attitude towards their children than that of their counterparts with healthy children [22]. In a study on illness perception focused on childhood cancer survivors and their caregivers, caregivers perceived the illness to be more severe compared to the perception of cancer survivors themselves; the study recommend the need for expert intervention to bridge this gap in perception [23]. Moreover, they emphasized that these perception gap findings would provide valuable insights into the understanding of relationships with other mental health variables, including psychological trauma, fatigue, and the process of returning to school and work [23]. They also stressed the importance of monitoring these differences in perception between survivors and caregivers to gain better understanding of the interconnections among these variables [23].

However, previous reports including that in ROK have highlighted the significance of various cancer survivor support programs. Physical support programs, such as those focusing on nutrition, exercise, and diet for adolescent cancer survivors, as well as psychological support programs such as counseling, are also crucial. Moreover, it is equally important to educate classmates and co-workers who will be part of the lives of adolescent cancer survivors.

There are several limitations that should be considered when interpreting the findings of this study. First, although the survey was conducted on a nationwide scale, it employed a non-probability sample with a proportional population allocated by sex, age, and region. Consequently, the generalizability of the survey results to the entire adolescent population may be constrained. However, given the challenge involved in conducting a face-to-face survey, and obtaining parental consent for participation, this study was deemed the most suitable approach. Second, this study is a survey conducted within one country; therefore, adolescent perspectives and preferences gathered from this study may have limited generalizability to the broader adolescent population. Finally, this study focused on understanding the current situation, and due to limitations, such as the number of survey questions, identifying the reasons and triggers for the participants’ perceptions of adolescent cancer survivors through qualitative research or additional survey items proved challenging. Including this type of research in the future could have provided valuable insights for developing effective and age-appropriate educational programs to promote healthy behaviors among adolescent cancer survivors.

## Conclusion

The perception of cancer among healthy adolescents was relatively accurate; however, when it came to interacting with adolescent cancer survivors, healthy adolescents exhibited dualistic thinking. These perceptions of healthy adolescents could impede the successful reintegration of adolescent cancer survivors into daily life. Therefore, it is crucial to implement comprehensive education programs for healthy adolescents about illness and cancer survivors, enabling them to interact and collaborate positively with cancer survivors in the academic and professional settings. Moreover, the findings of this study suggest the need for a comprehensive approach that incorporates educational policies for healthy adolescents alongside healthcare policies, rather than limiting support policies for adolescent cancer survivors to the realm of healthcare.

Further research is required, rigorously designed and conducted, to validate the challenges adolescent cancer survivors encounter when reintegrating into daily life and social activities. Additionally, exploring the relationship between healthy adolescents’ negative perceptions of interaction with adolescent cancer survivors in academic and workplace settings warrant further investigation.

### Supplementary Information

Supplementary Material 1.

## Data Availability

The datasets generated during and/or analyzed during the current study are available from the corresponding author on reasonable request.

## Abbreviations

ROK: Republic of Korea
IRB: Institutional Review Board
