# Supplementary material for: Perception of Korean healthy adolescents on cancer and adolescent cancer survivors: a cross-sectional survey
Source: BMC Public Health. 2024 Jun 26;24:1700. doi: 10.1186/s12889-024-19192-4 (PMC11202335; doi:10.1186/s12889-024-19192-4)
Supplement: Supplementary file 1 — Supplementary Material 1. [file 12889_2024_19192_MOESM1_ESM.docx]

**Appendix 1.** Number of survey participants secured by region

**
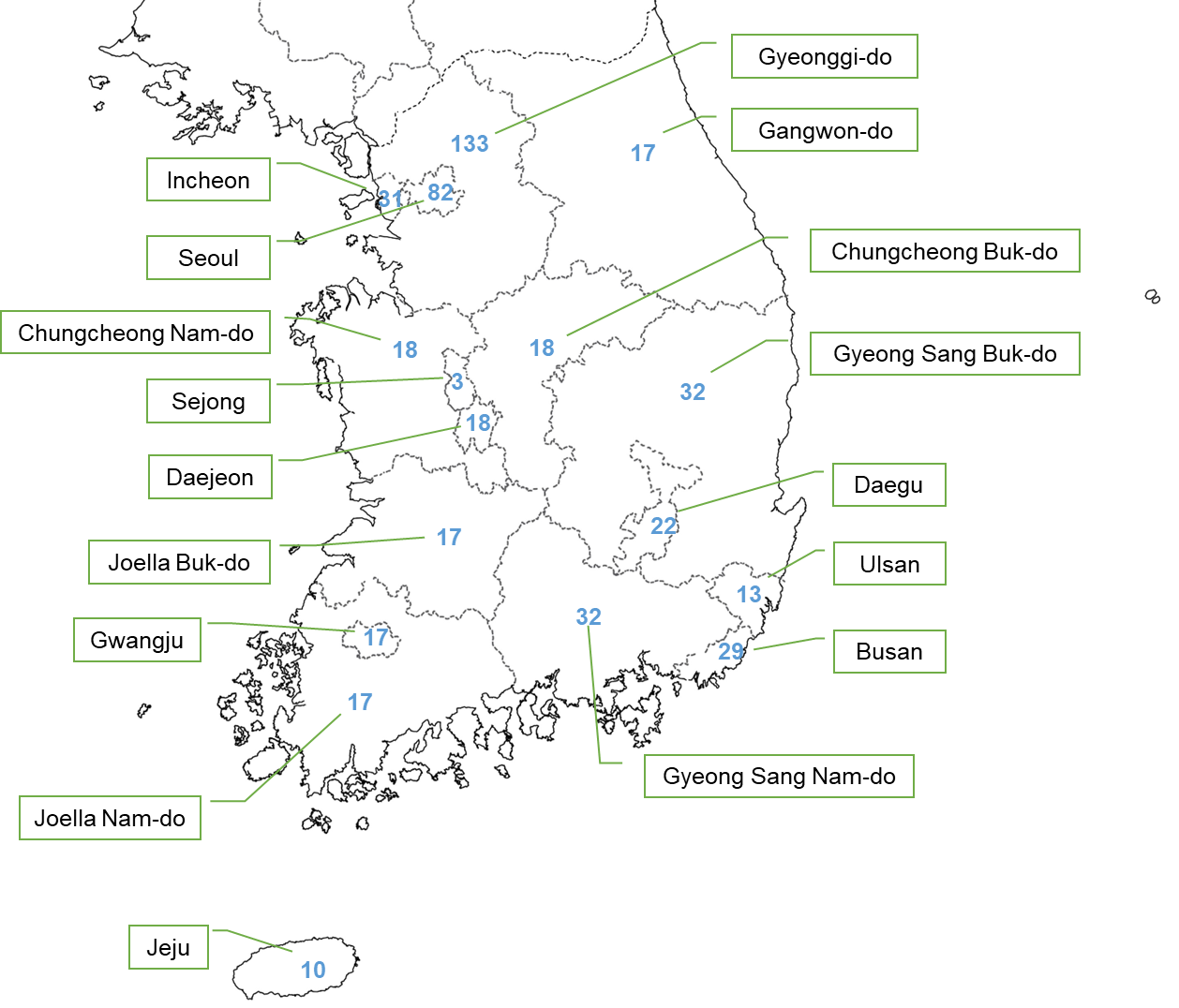
**

**Appendix 2.** Adolescents’ perception of cancer, asthma, measles, and tuberculosis

| Variables | | Caner | | Tuberculosis | |  | Measles | |  | Asthma | |  | *P*** |
| --- | --- | --- | --- | --- | --- | --- | --- | --- | --- | --- | --- | --- | --- |
|  |  | n | % | n | % | *P** | n | % | *P** | n | % | *P** |  |
| Contagious | |  |  |  |  |  |  |  |  |  |  |  |  |
|  | Yes | 42 | 8.42 | 367 | 86.76 | 0.141 | 331 | 78.25 | **0.012** | 331 | 78.25 | **<0.001** | **<0.001** |
|  |  |  |  |  |  |  |  |  |  |  |  |  |  |
| Affecting adolescents | |  |  |  |  |  |  |  |  |  |  |  |  |
|  | Yes | 331 | 66.33 | 366 | 86.52 | **<0.001** | 382 | 90.31 | **<0.001** | 348 | 76.65 | **<0.001** | **<0.001** |
|  |  |  |  |  |  |  |  |  |  |  |  |  |  |
| Preventable | |  |  |  |  |  |  |  |  |  |  |  |  |
|  | Yes | 361 | 72.34 | 365 | 86.29 | **0.003** | 316 | 74.70 | **<0.001** | 296 | 65.20 | **<0.001** | **<0.001** |
|  |  |  |  |  |  |  |  |  |  |  |  |  |  |
| Serious | |  |  |  |  |  |  |  |  |  |  |  |  |
|  | Yes | 475 | 95.19 | 297 | 70.21 | 0.539 | 206 | 48.70 | 0.104 | 296 | 65.20 | 0.178 | 0.334 |
|  |  |  |  |  |  |  |  |  |  |  |  |  |  |
| Curable | |  |  |  |  |  |  |  |  |  |  |  |  |
|  | Yes | 460 | 92.18 | 408 | 96.45 | 0.390 | 410 | 96.93 | 0.237 | 378 | 83.26 | 0.092 | 0.234 |
|  |  |  |  |  |  |  |  |  |  |  |  |  |  |

*Comparison with cancer; ** Comparison of cancer, tuberculosis, measles, and asthma

**Appendix 3.** Adolescents’ perception of cancer, asthma, measles, and tuberculosis by sex

| Variables | | Caner | | | | | Tuberculosis | | | | | Measles | | | | | Asthma | | | | |
| --- | --- | --- | --- | --- | --- | --- | --- | --- | --- | --- | --- | --- | --- | --- | --- | --- | --- | --- | --- | --- | --- |
|  |  | Male | | Female | | *P* | Male | | Female | | *P* | Male | | Female | | *P* | Male | | Female | | *P* |
|  |  | n | % | n | % |  | n | % | n | % |  | n | % | n | % |  | n | % | n | % |  |
| Contagious | |  |  |  |  |  |  |  |  |  |  |  |  |  |  |  |  |  |  |  |  |
|  | Yes | 25 | 9.62 | 17 | 7.11 | 0.315 | 197 | 87.95 | 170 | 85.43 | 0.445 | 183 | 80.26 | 148 | 75.90 | 0.278 | 208 | 86.31 | 170 | 79.81 | 0.064 |
|  |  |  |  |  |  |  |  |  |  |  |  |  |  |  |  |  |  |  |  |  |  |
| Affecting adolescents | |  |  |  |  |  |  |  |  |  |  |  |  |  |  |  |  |  |  |  |  |
|  | Yes | 167 | 64.23 | 164 | 68.62 | 0.300 | 197 | 87.95 | 169 | 84.92 | 0.364 | 209 | 91.67 | 173 | 88.72 | 0.307 | 176 | 73.03 | 172 | 80.75 | 0.052 |
|  |  |  |  |  |  |  |  |  |  |  |  |  |  |  |  |  |  |  |  |  |  |
| Preventable | |  |  |  |  |  |  |  |  |  |  |  |  |  |  |  |  |  |  |  |  |
|  | Yes | 193 | 74.23 | 168 | 70.29 | 0.326 | 187 | 83.48 | 178 | 89.45 | 0.075 | 170 | 74.56 | 146 | 74.87 | 0.942 | 162 | 67.22 | 134 | 62.91 | 0.336 |
|  |  |  |  |  |  |  |  |  |  |  |  |  |  |  |  |  |  |  |  |  |  |
| Serious | |  |  |  |  |  |  |  |  |  |  |  |  |  |  |  |  |  |  |  |  |
|  | Yes | 249 | 95.77 | 226 | 94.56 | 0.528 | 158 | 70.54 | 139 | 69.85 | 0.878 | 107 | 46.93 | 99 | 50.77 | 0.431 | 143 | 59.34 | 153 | 71.83 | **0.005** |
|  |  |  |  |  |  |  |  |  |  |  |  |  |  |  |  |  |  |  |  |  |  |
| Curable | |  |  |  |  |  |  |  |  |  |  |  |  |  |  |  |  |  |  |  |  |
|  | Yes | 238 | 91.54 | 222 | 92.89 | 0.575 | 218 | 97.32 | 190 | 95.48 | 0.306 | 223 | 97.81 | 187 | 95.90 | 0.257 | 208 | 86.31 | 170 | 79.81 | 0.064 |
|  |  |  |  |  |  |  |  |  |  |  |  |  |  |  |  |  |  |  |  |  |  |

**Appendix 4.** Adolescents’ perception of cancer, asthma, measles, and education stage

| Variables | | Caner | | | | | Tuberculosis | | | | | Measles | | | | | Asthma | | | | |
| --- | --- | --- | --- | --- | --- | --- | --- | --- | --- | --- | --- | --- | --- | --- | --- | --- | --- | --- | --- | --- | --- |
|  |  | Middle | | High | | *P* | Middle | | High | | *P* | Middle | | High | | *P* | Middle | | High | | *P* |
|  |  | n | % | n | % |  | n | % | n | % |  | n | % | n | % |  | n | % | n | % |  |
| Contagious | |  |  |  |  |  |  |  |  |  |  |  |  |  |  |  |  |  |  |  |  |
|  | Yes | 21 | 10.82 | 21 | 6.89 | 0.122 | 129 | 83.23 | 238 | 88.81 | 0.103 | 116 | 74.36 | 215 | 80.52 | 0.138 | 36 | 20.93 | 38 | 13.48 | **0.037** |
|  |  |  |  |  |  |  |  |  |  |  |  |  |  |  |  |  |  |  |  |  |  |
| Affecting adolescents | |  |  |  |  |  |  |  |  |  |  |  |  |  |  |  |  |  |  |  |  |
|  | Yes | 135 | 69.59 | 196 | 64.26 | 0.220 | 133 | 85.81 | 233 | 86.94 | 0.742 | 142 | 91.03 | 240 | 89.89 | 0.703 | 140 | 81.40 | 208 | 73.76 | 0.062 |
|  |  |  |  |  |  |  |  |  |  |  |  |  |  |  |  |  |  |  |  |  |  |
| Preventable | |  |  |  |  |  |  |  |  |  |  |  |  |  |  |  |  |  |  |  |  |
|  | Yes | 151 | 77.84 | 210 | 68.85 | **0.029** | 124 | 80.00 | 241 | 89.93 | **0.004** | **126** | 80.77 | 190 | 71.16 | **0.028** | 118 | 68.60 | 178 | 63.12 | 0.234 |
|  |  |  |  |  |  |  |  |  |  |  |  |  |  |  |  |  |  |  |  |  |  |
| Serious | |  |  |  |  |  |  |  |  |  |  |  |  |  |  |  |  |  |  |  |  |
|  | Yes | 186 | 95.88 | 289 | 94.75 | 0.568 | 118 | 76.13 | 179 | 66.79 | **0.043** | 75 | 48.08 | 131 | 49.06 | 0.845 | 124 | 72.09 | 172 | 60.99 | **0.016** |
|  |  |  |  |  |  |  |  |  |  |  |  |  |  |  |  |  |  |  |  |  |  |
| Curable | |  |  |  |  |  |  |  |  |  |  |  |  |  |  |  |  |  |  |  |  |
|  | Yes | 174 | 89.69 | 286 | 93.77 | 0.098 | 148 | 95.48 | 260 | 97.01 | 0.412 | 152 | 97.44 | 258 | 96.63 | 0.643 | 141 | 81.98 | 237 | 84.04 | 0.567 |
|  |  |  |  |  |  |  |  |  |  |  |  |  |  |  |  |  |  |  |  |  |  |

**Appendix 5.** Health information resources

| Variables | Caner | | Tuberculosis | |  | Measles | |  | Asthma | |  | *P*** |
| --- | --- | --- | --- | --- | --- | --- | --- | --- | --- | --- | --- | --- |
|  | n | % | n | % | *P** | n | % | *P** | n | % | *P** |  |
| Television | 212 | **42.40** | 176 | 35.20 | **<0.001** | 151 | 30.20 | **<0.001** | 185 | 37.00 | **<0.001** | **<0.001** |
| Internet | 233 | **46.60** | 139 | 27.80 | **<0.001** | 147 | 29.40 | **<0.001** | 181 | 36.20 | **<0.001** | **<0.001** |
| Book | 30 | **6.00** | 60 | 12.00 | **<0.001** | 51 | 10.20 | **<0.001** | 39 | 7.80 | **<0.001** | **<0.001** |
| Teacher | 38 | **7.60** | 58 | 11.60 | **<0.001** | 58 | 11.60 | **<0.001** | 28 | 5.60 | **<0.001** | **<0.001** |
| Parents | 40 | **8.00** | 32 | 6.40 | **<0.001** | 37 | 7.40 | **<0.001** | 33 | 6.60 | **<0.001** | **<0.001** |
| Siblings | 3 | **0.60** | 2 | 0.40 | 0.912 | 4 | 0.80 | **<0.001** | 5 | 1.00 | **<0.001** | **<0.001** |
| Friends | 9 | **1.80** | 10 | 2.00 | **<0.001** | 11 | 2.20 | **<0.001** | 12 | 2.40 | **<0.001** | **<0.001** |

Note.Multiple responses available

*Comparison with cancer; ** Comparison of cancer, tuberculosis, measles, and asthma

**Appendix 6.** Health information resources by sex

| Variables | | Caner | | | | | Tuberculosis | | | | | Measles | | | | | Asthma | | | | |
| --- | --- | --- | --- | --- | --- | --- | --- | --- | --- | --- | --- | --- | --- | --- | --- | --- | --- | --- | --- | --- | --- |
|  |  | Male | | Female | | *P* | Male | | Female | | *P* | Male | | Female | | *P* | Male | | Female | | *P* |
|  |  | n | % | n | % |  | n | % | n | % |  | n | % | n | % |  | n | % | n | % |  |
| Television | | 99 | 38.08 | 113 | 47.08 | **0.042** | 92 | 35.38 | 84 | 35.00 | 0.928 | 73 | 28.08 | 78 | 32.50 | 0.282 | 94 | 36.15 | 91 | 37.92 | 0.683 |
| Internet | | 136 | 52.31 | 97 | 40.42 | **0.008** | 84 | 32.31 | 55 | 22.92 | **0.019** | 95 | 36.54 | 52 | 21.67 | **<0.001** | 109 | 41.92 | 72 | 30.00 | **0.006** |
| Book | | 15 | 5.77 | 15 | 6.25 | 0.821 | 27 | 10.38 | 33 | 13.75 | 0.247 | 26 | 10.00 | 25 | 10.42 | 0.878 | 21 | 8.08 | 18 | 7.50 | 0.810 |
| Teacher | | 16 | 6.15 | 22 | 9.17 | 0.204 | 25 | 9.62 | 33. | 13.75 | 0.149 | 30 | 11.54 | 28 | 11.67 | 0.964 | 14 | 5.38 | 14 | 5.38 | 0.827 |
| Parents | | 23 | 8.85 | 17 | 7.08 | 0.468 | 14 | 5.38 | 18 | 7.50 | 0.334 | 21 | 8.08 | 16 | 6.67 | 0.547 | 15 | 5.77 | 18 | 7.50 | 0.436 |
| Siblings |  | 1 | 0.38 | 2 | 0.83 | 0.516 | 0 | 0 | 2 | 0.83 | 0.140 | 1 | 0.38 | 3 | 1.25 | 0.278 | 1 | 0.38 | 4 | 1.67 | 0.150 |
| Friends |  | 4 | 1.54 | 5 | 2.08 | 0.647 | 5 | 1.92 | 5 | 2.08 | 0.898 | 6 | 2.31 | 5 | 2.08 | 0.864 | 7 | 2.69 | 5 | 2.08 | 0.657 |

**Appendix 7.** Health information resources by education stage

| Variables | | Caner | | | | | Tuberculosis | | | | | Measles | | | | | Asthma | | | | |
| --- | --- | --- | --- | --- | --- | --- | --- | --- | --- | --- | --- | --- | --- | --- | --- | --- | --- | --- | --- | --- | --- |
|  |  | Middle | | High | | *P* | Middle | | High | | *P* | Middle | | High | | *P* | Middle | | High | | *P* |
|  |  | n | % | n | % |  | n | % | n | % |  | n | % | n | % |  | n | % | n | % |  |
| Television | | 79 | 40.72 | 133 | 43.46 | 0.545 | 62 | 31.96 | 114 | 37.25 | 0.227 | 42 | 21.65 | 109 | 35.62 | **0.001** | 60 | 30.93 | 125 | 40.85 | **0.025** |
| Internet | | 87 | 44.85 | 146 | 47.71 | 0.531 | 56 | 28.87 | 83 | 27.12 | 0.672 | 62 | 31.96 | 85 | 27.78 | 0.317 | 71 | 36.60 | 110 | 35.95 | 0.883 |
| Book | | 12 | 6.19 | 18 | 5.88 | 0.889 | 20 | 10.31 | 40 | 13.07 | 0.354 | 22 | 11.34 | 29 | 9.48 | 0.502 | 20 | 10.31 | 19 | 6.21 | 0.096 |
| Teacher | | 10 | 5.15 | 28 | 9.15 | 0.100 | 16 | 8.25 | 42 | 13.73 | 0.062 | 22 | 11.34 | 36 | 11.76 | 0.885 | 8 | 4.12 | 20 | 6.54 | 0.253 |
| Parents | | 20 | 10.31 | 20 | 6.54 | 0.130 | 12 | 6.19 | 20 | 6.54 | 0.876 | 14 | 7.22 | 23 | 7.52 | 0.901 | 12 | 6.19 | 21 | 6.86 | 0.766 |
| Siblings |  | 1 | 0.52 | 2 | 0.65 | 0.845 | 1 | 0.52 | 1 | 0.33 | 0.745 | 2 | 1.03 | 2. | 0.65 | 0.644 | 2 | 1.03 | 3. | 0.98 | 0.956 |
| Friends |  | 6 | 3.09 | 3 | 0.98 | 0.083 | 4 | 2.06 | 6 | 1.96 | 0.937 | 5 | 2.58 | 6 | 1.96 | 0.647 | 6 | 3.09 | 6 | 1.96 | 0.420 |
